# Cerebrospinal fluid kynurenine and kynurenic acid concentrations are associated with coma duration and long-term neurocognitive impairment in Ugandan children with cerebral malaria

**DOI:** 10.1186/s12936-017-1954-1

**Published:** 2017-07-28

**Authors:** Dag Holmberg, Elisabeth Franzén-Röhl, Richard Idro, Robert O. Opoka, Paul Bangirana, Carl M. Sellgren, Ronny Wickström, Anna Färnert, Lilly Schwieler, Göran Engberg, Chandy C. John

**Affiliations:** 1 Department of Physiology & Pharmacology, Karolinska Institutet, Stockholm, Sweden; 2 Department of Medicine Solna, Unit of Infectious Diseases, Karolinska Institutet, Stockholm, Sweden; 3 Department of Infectious Diseases, Karolinska University Hospital, Stockholm, Sweden; 4 Department of Paediatrics and Child Health, Makerere University, Kampala, Uganda; 5 Department of Psychiatry, Makerere University, Kampala, Uganda; 6 Stanley Center for Psychiatric Research, Broad Institute of MIT and Harvard, Cambridge, MA USA; 7 Department of Women’s and Children’s Health, Karolinska Institutet, Stockholm, Sweden; 8 Department of Pediatrics, Indiana University, Indianapolis, IN USA; 9 Department of Pediatrics, University of Minnesota, Minnesota, USA

**Keywords:** Cerebral malaria, Kynurenine, Kynurenic acid, Cognition, Coma, Cytokines, INF-γ, TNF

## Abstract

**Background:**

One-fourth of children with cerebral malaria (CM) retain cognitive sequelae up to 2 years after acute disease. The kynurenine pathway of the brain, forming neuroactive metabolites, e.g. the NMDA-receptor antagonist kynurenic acid (KYNA), has been implicated in long-term cognitive dysfunction in other CNS infections. In the present study, the association between the kynurenine pathway and neurologic/cognitive complications in children with CM was investigated.

**Methods:**

Cerebrospinal fluid (CSF) concentrations of KYNA and its precursor kynurenine in 69 Ugandan children admitted for CM to Mulago Hospital, Kampala, Uganda, between 2008 and 2013 were assessed. CSF kynurenine and KYNA were compared to CSF cytokine levels, acute and long-term neurologic complications, and long-term cognitive impairments. CSF kynurenine and KYNA from eight Swedish children without neurological or infectious disease admitted to Astrid Lindgren’s Children’s Hospital were quantified and used for comparison.

**Results:**

Children with CM had significantly higher CSF concentration of kynurenine and KYNA than Swedish children (*P* < 0.0001 for both), and CSF kynurenine and KYNA were positively correlated. In children with CM, CSF kynurenine and KYNA concentrations were associated with coma duration in children of all ages (*P* = 0.003 and 0.04, respectively), and CSF kynurenine concentrations were associated with worse overall cognition (*P* = 0.056) and attention (*P* = 0.003) at 12-month follow-up in children ≥5 years old.

**Conclusions:**

CSF KYNA and kynurenine are elevated in children with CM, indicating an inhibition of glutamatergic and cholinergic signaling. This inhibition may lead acutely to prolonged coma and long-term to impairment of attention and cognition.

## Background

Cerebral malaria (CM) is estimated to occur in as many as 500,000 children a year, with a high mortality and post-discharge morbidity [1]. CM is characterized by coma that typically occurs 1–3 days after fever onset [1]. Although the mechanisms that lead to the clinical presentation of CM are not entirely understood, sequestration of infected erythrocytes in cerebral capillaries and post-capillary venules and inflammatory responses in the central nervous system (CNS) both appear to play a role [2]. Cognitive impairment after CM is a well-described complication and affects 14–25% of the children at least for several years after the episode [3–7]. CNS inflammation may contribute to brain damage as well as the neurocognitive sequelae of CM [1, 8, 9].

**Fig. 1:**
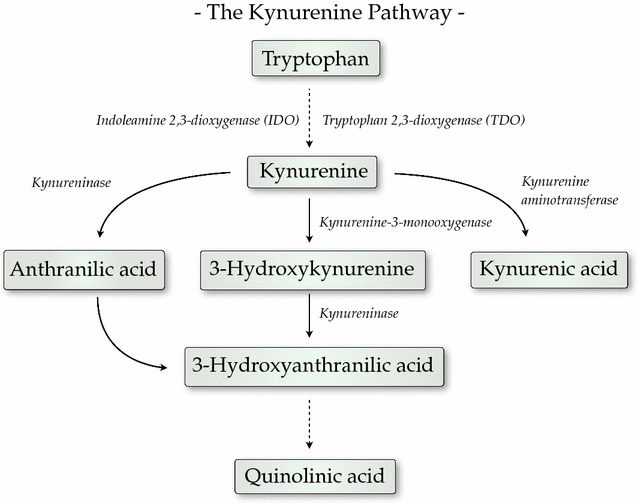
The kynurenine pathway of tryptophan degradation

Tryptophan degradation along the kynurenine pathway is largely regulated by cytokines and give rise to the neuroactive kynurenic acid (KYNA), a compound that blocks the glycine co-agonist site of the *N*-methyl-d-aspartic acid receptor (NMDAR) as well as the cholinergic α7 nicotinic receptor (α7nAChR) [10] (Fig. 1). NMDAR, as well as α7nAChR, have an established role in cognitive functions, [11–13] and animal studies show that increased brain KYNA is associated with cognitive deficits [10, 11]. Moreover, KYNA is elevated in the cerebrospinal fluid (CSF) after CNS infections and in psychotic disorders [14–16]. Thus, KYNA could influence acute disease as well as long-term cognitive sequelae in CM [17, 18]. Another metabolite of the kynurenine pathway, the neurotoxic quinolinic acid is also elevated in the cerebrospinal fluid CSF in children with CM, indicating that a more general activation of the kynurenine pathway is involved in the disease [19].

Brain KYNA was hypothesized to be increased in children with CM, which could possibly exert acute effects similar to that of the anesthetic NMDAR antagonist ketamine (loss of consciousness), in addition to possible long-term effects on cognition. To evaluate this, kynurenine and KYNA concentrations was assessed in CSF of Ugandan children presenting with CM and correlated this to acute neurologic outcomes, including coma duration, number of seizures and presence of gross neurologic deficits at discharge, and to the long-term neurocognitive outcomes of overall cognitive ability and attention 12 months after the episode of CM.

## Methods

### Study design

The children in this study were part of a larger study assessing long-term neurocognitive outcomes in children with cerebral malaria and severe malarial anaemia in community children. A detailed description of the study cohort has been published previously [20]. Briefly, children with CM between 18 months and 12 years of age were enrolled at Mulago Hospital, Kampala, Uganda from 2008 to 2013. Cerebral malaria was defined as: (1) coma (Blantyre Coma Scale [BCS] score ≤2 in children ≤5 years; or Glasgow Coma Scale [GCS] score ≤8 in children >5 years); (2) *Plasmodium falciparum* on blood smear; and (3) no other known cause of coma (e.g., meningitis, a prolonged postictal state or hypoglycaemia-associated coma reversed by glucose infusion). Children with CM were managed according to the current Ugandan Ministry of Health treatment guidelines at the time of the study. These included intravenous quinine followed by oral artemisinin-based combination treatment for outpatient follow-up therapy, and lumbar puncture to obtain CSF to rule out bacterial meningitis or encephalitis, unless the procedure was clinically contraindicated or unless parents refused the procedure. Children with either (1) known chronic illness requiring medical care; (2) known developmental delay; or (3) prior history of coma, head trauma, hospitalization for malnutrition, or cerebral palsy were excluded from the study. Informed consent was obtained from parents or guardians of study participants. 269 children with CM were enrolled in the study, of whom 69 had sufficient CSF sample to be tested for kynurenine and KYNA levels after aliquots were made for other testing, including pro- and anti-inflammatory cytokine testing and testing for nitric oxide levels. Results from the 69 children who had CSF available for kynurenine and KYNA testing are presented in this paper.

Since CSF cannot be ethically obtained from a control group of otherwise healthy Ugandan children, CSF KYNA and kynurenine were compared to CSF from age-matched children (n = 8) admitted to Astrid Lindgren Children’s Hospital in Stockholm, Sweden. The children in this comparison group were admitted due to diffuse neurological symptoms and underwent lumbar puncture in the acute phase for differential diagnostic purposes. Two children presented with seizures due to high fever, one with viral gastroenteritis, one with rhinovirus infection, one with syncope, one with neurological deficits from the posterior circulation, and finally two with unclear diagnosis but with no indication of CNS disease. No children were diagnosed with CNS infections. Ethical approvals were granted by the Institutional Review Boards for Human Studies at Makerere University School of Medicine, University of Minnesota, and Michigan State University, as well as by the Regional Ethical Committee in Stockholm.

### Clinical and demographic assessment

Children with CM underwent a medical history and physical examination and were assessed for malaria retinopathy (21) by indirect ophthalmoscopy. Nutrition was assessed by height and weight for age z-scores (Epi Info v. 3.5.3, CDC, Atlanta GA). Duration of coma was defined as the time period from admission in coma to full resolution of coma (time to BCS = 5 or GCS = 15).

### Analysis of kynurenine and KYNA

In order to quantify CSF KYNA and kynurenine, an isocratic reverse-phase HPLC system, including a dual-piston, liquid delivery pump (LC 10 AD Shimadzu, Japan) and a ReproSil-Pur C18 column (4 × 100 mm, Dr. Maisch GmbH, Ammerbuch, Germany) was used [21]. Samples of 30 µL CSF were manually injected and eluted with a mobile phase of 50 mmol/L sodium acetate (pH 6.20) and 7% acetonitrile (flow rate of 0.5 mL/min). Kynurenine was detected with a UV detector at 360 nm. A 0.5 mol/L zinc acetate solution was then added to the eluate at a flow rate of 10 mL/h with a P-500 pump (Pharmacia, Uppsala, Sweden). KYNA was then detected using a fluorescence detector (Jasco Ltd, Hachiohji City, Japan) with excitation wavelength of 344 nm and emission wavelength of 398 nm. Approximate retention time for KYNA and kynurenine was 7.8 and 4.2 min, respectively.

HPLC fluorescent/UV emissions were analysed using Datalys Azur, version 4.6.0.0 (DATALYS, Saint-Martin-d’Hères, France). Using linear equation and standard mixtures of KYNA (0.16–60 nM) and kynurenine (0.16–10 nM), the voltages obtained from the detectors were then translated to reciprocal nanomolar values via Prism 6 programs (GraphPad Software). KYNA concentrations >1000 nM were considered unreliable and diluted 1/10 for confirming analysis. CSF interferon (IFN)-γ and tumour necrosis factor (TNF) levels were measured by ELISA [22] and cytometric bead assay [2] respectively, as previously described.

### Cognitive assessment

Children were tested for cognitive function a week after discharge and then 6 and 12 months after enrollment. The primary study endpoint was cognitive function 12 months after enrollment (long-term cognitive function). For children <5 years of age, the Mullen scales of early learning [23] were used to measure cognitive ability. Scores from fine motor, visual reception, receptive language, and expressive language scales were summed to give the early learning composite score, a measure of overall cognitive ability. Attention was assessed using the early childhood vigilance test [24]. For children ≥5 years of age, the Kaufman assessment battery for children-II (KABC-II) was used to test for overall cognitive ability, with scores from subtests summed to provide a mental processing index score [3]. The test of variables of attention was used to assess attention, with the D prime score as the primary outcome variable [3]. Results for these tests in Ugandan children with cerebral malaria versus community children were described in an earlier study cohort [3]. Cognitive testing was not performed in the comparison group.

### Statistical analysis

CSF kynurenine and KYNA levels in children with CM and the comparison group were compared using Mann–Whitney U test. Age-adjusted z-scores for cognitive outcomes were created using the scores of Ugandan community children as previously described [20].

Spearman’s rank correlation analysis was used to compare CSF levels of kynurenine and KYNA, and clinical factors and CSF cytokine levels to CSF kynurenine or KYNA levels. Linear regression, logistic regression and negative binomial regression analyses were used to compare KYNA and kynurenine concentrations to continuous, categorical and count outcomes, respectively. Analysis with KYNA and kynurenine concentrations were adjusted for age and lactate. Analyses were conducted in Stata 14 (Stata Corporation, College Station, TX) and SPSS 23 (SPSS Corporation, Chicago, IL).

## Results

### Demographic and clinical characteristics of children with CM

Sixty-nine children with CM (69.6% males) with a median age of 3.3 years [interquartile range (IQR), 2.5–4.4 years] were included in the study (Table 1). The included children had slightly longer coma duration, a slightly lower percentage of females, and higher levels of CSF IFN-γ and TNF compared to the full study cohort (Table 1). The mortality rate among the included children was 4.3% (3 of 69 children). Most children with CM had seizures at some point during their illness (92.7%), and the number of seizures ranged between 0 and 21. Median total coma duration was 54 h (IQR 40–77 h) (Table 1). Neurologic deficits were common at discharge, but had largely resolved by 12-month follow-up (Table 1). Two children with CM were co-infected with human immunodeficiency virus (HIV). The children of the comparison group were predominantly females (62.5%) with a median age of 1.4 years (IQR 3.6 months to 2.6 years).

**Table 1:** Demographic and clinical characteristics of Ugandan children with cerebral malaria with versus without cerebrospinal fluid (CSF) kynurenic acid (KYNA) measurement

Characteristic	Included children (N = 69^a^)	Excluded children (N = 200^a^)	P value^c^
Age, years, median (inter-quartile range)	3.3 (2.5, 4.4)	3.5 (2.5, 4.9)	0.71
Sex, n, (% female)	21 (30.4%)	89 (44.5%)	0.04
Hemoglobin, g/dL, mean (SD)	7.0 (2.6)	6.9 (2.2)	0.72
Parasitaemia, parasites/µL, median (IQR) (N)	44,280 (12,920, 294,660) (65)	48,420 (10,880, 262,060) (197)	0.96
Lactic acidosis present^b^, n/N (%)	20/65 (30.8%)	69/185 (37.3%)	0.34
Malaria retinopathy present, n/N (%)	43/68 (63.2%)	124/187 (66.3)	0.64
Convulsions during illness (yes/no), n (%)	64 (92.7%)	187 (93.5%)	0.83
Number of convulsions in hospital, median (IQR)	1 (0, 2)	1 (0, 2)	0.37
Total hours in coma, median (IQR) (N)	54 (40, 77) (66)	44 (18.25, 80) (193)	0.04
Neurologic deficits at discharge, n/N (%)	27/66 (40.9%)	56/166 (33.7%)	0.3
Neurologic deficits, 12 mo follow-up, n/N (%)	2/62 (3.2%)	5/161 (3.1%)	0.96
CSF IFN-γ, pg/mL, median (IQR) (N)	30.3 (16.1, 47.8) (52)	20.5 (11.8, 26.1) (94)	0.004
CSF TNF-α, pg/mL, median (IQR) (N)	1.8 (0.8, 3.3) (69)	1.2 (0.1, 2.4) (110)	0.02

*IFN* interferon, *TNF* tumour necrosis factor

^a^For variables for which N is less than the total N listed for group, N’s for that variable and group are noted in the table

^b^Lactate value >5 µmol/L

^c^Wilcoxon rank-sum test for continuous variables, χ^2^ test for categorical variables

### CSF kynurenine and KYNA concentrations in children with CM

**Fig. 2:**
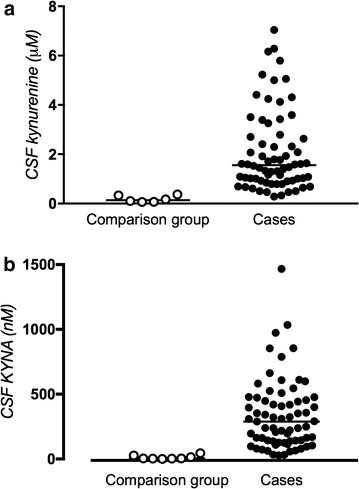
**a** CSF kynurenine concentrations in children diagnosed with cerebral malaria versus comparison group. Children diagnosed with CM (n = 69) had significant increases in CSF kynurenine (*P* < 0.001, Mann–Whitney U test). *Symbols* represent CSF kynurenine concentration in individual children. *Horizontal line* shows median value. **b** CSF KYNA concentrations in children diagnosed with cerebral malaria versus comparison group. Children diagnosed with CM (n = 69) had significantly increased CSF KYNA compared to a Swedish comparison group (n = 8) (*P* < 0.001, Mann–Whitney U test). *Symbols* represent CSF KYNA concentration in individual children. *Horizontal line* show median value

CSF kynurenine concentrations in children with CM (median 1555.0 nM [IQR 927.5–2722.5]) were significantly higher than in the comparison group (median 134.5 nM [IQR 72.3–289.8]; *P* < 0.001; Fig. 2a). CSF KYNA concentrations in children with CM (median 289.4 nM [IQR 137.2–463.7]) were also significantly higher than in the comparison group (median 3.8 nM [IQR 3.5–17.3]; *P* < 0.001; Fig. 2b). CSF KYNA and CSF kynurenine values were strongly positively correlated (Spearman’s rho = 0.7; *P* < 0.001). CSF KYNA and kynurenine concentrations did not differ significantly in the two HIV-infected children compared to the HIV negative children (P = 0.81). Median serum creatinine concentration was 0.4 [IQR 0.3–0.5].

### CSF kynurenine and KYNA concentrations, demographic and clinical factors, and CSF cytokine concentrations

Among demographic and clinical factors, age was negatively correlated with KYNA and kynurenine (Spearman’s rho = −0.5, *P* = 0.0001, and −0.3, *P* = 0.004, respectively, Table 2a, b). CSF kynurenine was also associated with blood lactate level (Table 2b). Further, CSF KYNA and kynurenine concentrations were positively correlated to CSF TNF (Spearman’s rho = 0.3, *P* = 0.03, and 0.30, *P* = 0.01, respectively; Table 2a, b). In contrast, CSF IFN-γ was not correlated to neither CSF kynurenine nor KYNA (Table 2a, b). Moreover, CSF KYNA and CSF kynurenine were not associated with serum creatinine (Spearman’s rho = 0.1, *P* = 0.31 and 0.2, *P* = 0.08, respectively).

**Table 2:** Relationship of demographic and clinical characteristics and cerebrospinal fluid (CSF) cytokine concentrations in 69 Ugandan children with cerebral malaria to CSF (a) KYNA and (b) kynurenine concentration

Characteristic	Spearman’s rho value (95% CI)	*P*
a		
Age	−0.45 (−0.62,−0.24)	0.0001
Sex	−0.06 (−0.29, 0.18)	0.63
Parasite density	−0.01 (−0.25, 0.24)	0.94
Lactate level	0.14 (−0.11, 0.37)	0.26
Malaria retinopathy	0.15 (−0.09, 0.37)	0.23
CSF IFN-γ	0.22 (−0.07, 0.47)	0.12
CSF TNF	0.27 (0.03, 0.48)	0.03
b		
Age	−0.34 (−0.53, −0.11)	0.004
Sex	−0.003 (−0.24, 0.24)	0.98
Parasite density	−0.09 (−0.33, 0.16)	0.46
Lactate level	0.26 (0.01, 0.47)	0.04
Malaria retinopathy	0.06 (−0.18, 0.30)	0.61
CSF IFN-γ	0.22 (−0.06, 0.48)	0.11
CSF TNF	0.30 (0.05, 0.50)	0.01

### CSF kynurenine and KYNA results according to presence of retinopathy

Median CSF kynurenine and KYNA did not differ between children with no retinal changes on ophthalmoscopy (n = 25) (kynurenine: 1.5 nM [IQR 0.9–2.3], KYNA: 232.2 nM [IQR 95.1–442.8]) compared to children with malaria retinopathy (n = 42) (kynurenine: 1.6 nM [IQR 0.93–3.39], KYNA: 291.3 nM [IQR 163.0–477.3] (*P* = 0.61 and *P* = 0.22, respectively).

### CSF kynurenine and KYNA concentrations and clinical outcomes

Age-adjusted CSF kynurenine and KYNA concentrations were associated with coma duration (β = 9.1, 95% CI 4.0–14.3, *P* = 0.003 and β = 0.04, 95% CI 0.01–0.08, *P* = 0.04, respectively) (Table 3a, b). Neither of the metabolites were associated with mortality, number of seizures while hospitalized or neurological deficits at discharge or 12-month follow-up (Table 3a, b). Median KYNA concentrations were 291.3 [IQR 163.0–478.7] nM (n = 37), 419.7 [IQR 308.7–477.3] nM (n = 13) and 787.4 nM (n = 1) among children with BCS score equal to 2, 1 and 0, respectively (*P* = 0.06). CSF kynurenine concentrations were not associated with BCS score at admission (*P* = 0.22).

**Table 3:** Association of CSF (a) KYNA and (b) kynurenine with clinical outcomes in 69 Ugandan children with cerebral malaria

Clinical outcome	Value	Odds ratio, rate ratio or risk difference (95% CI)^a^	*P*
a			
Mortality	OR	1.00 (0.99, 1.01)	0.60
Neurologic deficits at discharge	OR	1.00 (1.00, 1.001)	0.55
Neurologic deficits, 6 mo follow-up	OR	0.99 (0.987, 1.002)	0.17
Number of convulsions in hospital	IRR	1.00 (0.999, 1.000)	0.88
Coma duration	β	0.04 (0.01, 0.08)	0.04
b			
Mortality	OR	1.49 (0.70, 3.16)	0.30
Neurologic deficits at discharge	OR	1.09 (0.79, 1.49)	0.61
Neurologic deficits, 6 mo follow-up	OR	0.99 (0.49, 1.99)	0.97
Number of convulsions in hospital	IRR	1.09 (0.97, 1.24)	0.15
Coma duration	β	9.1 (4.0, 14.3)	0.003

*OR* odds ratio, *IRR* incidence rate ratio, *95% CI* 95% confidence interval

^a^Logistic regression (OR), negative binomial regression (IRR) or multiple linear regression (β coefficient), adjusted for age and lactate level

### CSF kynurenine and KYNA concentrations and long-term neurocognitive outcomes

Cognition was assessed with different testing batteries in children <5 years of age and children ≥5 years of age, as outlined in the “Methods”. For this reason, assessment of neurocognitive outcome was analysed separately in the two age groups.

CSF kynurenine and KYNA were not associated with overall cognitive ability or attention z-scores at 12-month follow-up in children <5 years of age. However, at 12-month follow-up in children ≥5 years of age, log_10_ CSF kynurenine and KYNA concentrations were correlated negatively with attention (β = −2.5, 95% CI −4.0 to (−1.1); *P* = 0.003 and β = −2.1, 95% CI −4.9 to (−0.2); *P* = 0.03, respectively, Table 4a, b). In addition, in children ≥5 years old, CSF kynurenine concentrations correlated negatively to overall cognitive ability at 12-month follow-up, with a *P* value just above the standard value for significance (β = −1.8, 95% CI −3.7 to 0.05; P = 0.056, Table 4b).

**Table 4:** Relationship of (a) KYNA and (b) kynurenine levels with cognitive outcomes at 12-month follow-up in Ugandan children with cerebral malaria

Cognitive outcome	β coefficient (95% CI)^a^	P value
a		
Age <5 years, n = 40		
Overall cognitive ability	−0.6 (−2.6, 1.5)	0.57
Attention	−0.2 (−0.7, 1.2)	0.60
Age ≥5 years, n = 22		
Overall cognitive ability	−0.5 (−2.8, 1.8)	0.63
Attention	−2.1 (−4.9, −0.2)	0.03
b		
Age <5 years, n = 40		
Overall cognitive ability	−0.3 (−2.5, 2.0)	0.82
Attention	−0.5 (−1.5, 0.5)	0.29
Age ≥5 years, n = 22		
Overall cognitive ability	−1.8 (−3.7, 0.05)	0.056
Attention	−2.5 (−4.0, −1.1)	0.003

Log-transformed (base 10)

^a^Multiple linear regression, adjusted for age and lactate level

## Discussion

In the present study, the endogenous NMDAR antagonist KYNA and its immediate precursor kynurenine were markedly elevated in the CSF of children with CM, a finding consistent with previous reports suggesting an activation of the kynurenine pathway in CM [25–29]. CSF KYNA and kynurenine were also predictors of coma duration, and both metabolites were associated to long-term neurocognitive function in the area of attention. Moreover, the association between CSF kynurenine and KYNA concentrations observed in the present study are in line with previous observations [30] and indicate that the formation of KYNA is highly dependent on the availability of kynurenine.

Previous studies on CM with regard to kynurenine pathway metabolites have been somewhat conflicting. A study on Malawian children with CM [26] reported elevated CSF KYNA concentrations, consistent with the data in the present study. In contrast, a study on Vietnamese adults with severe malaria (80% of whom had CM) showed no difference in CSF KYNA concentrations compared to controls [25]. However, for the latter study, the observed levels of CSF KYNA in controls was 70-fold higher compared to previously reported concentrations in healthy adult volunteers [16, 31]. In the present study, CSF KYNA concentrations in children with CM were substantially higher in the children with CM as compared to an age-matched comparison group. A limitation of the present study was the comparison group of children, which were all collected from Sweden, with a genetic and social background different from the Ugandan children.

Notably, age was negatively correlated with kynurenine and KYNA. This is in contrast to the general view of an age-related increase in KYNA CSF observed in adult controls and neurologic, psychiatric, or infectious patients [31–33]. The observed negative correlation could be influenced by back length or body height of the children, previously found to be of importance for CSF KYNA concentration [34]. Back length and body height may also account for the slightly higher levels of kynurenine or KYNA seen in the comparison group when compared to adult controls [35].

The activity of the kynurenine pathway is strongly influenced by immune activation [10]. Thus, pro-inflammatory cytokines are known to induce the enzymes of the kynurenine pathway, most importantly tryptophan 2,3-dioxygenase (TDO) and indoleamine 2,3-dioxygenase (IDO), both being rate-limiting enzymes of this pathway [36, 37]. Previous experimental [38] and human studies [2] have shown an increase in intracerebral production of pro-inflammatory cytokines in CM. Numerous reports show that TNF or IFN-γ activate the kynurenine pathway [39] and given the observed positive correlation between CSF TNF and CSF kynurenine or KYNA in the present study, it is likely that TNF elevates kynurenine formation with a subsequent overflow in the KYNA branch of the kynurenine pathway in CM (Fig. 1).

A major finding of the study was the strong association between increased CSF KYNA and kynurenine to a prolonged coma duration. Indeed, KYNA shares pharmacological and behavioural features with the NMDAR antagonist ketamine, a frequently used anesthetic drug. At low concentrations, ketamine does not induce unconsciousness but a mere dissociative state in which the patient remains oblivious of surroundings [40]. In higher doses ketamine induces a higher state of consciousness with full surgical anesthesia. Given the presently disclosed correlation between CSF KYNA and coma duration, it is possible that the increased CSF KYNA concentration seen in the acute phase of CM induces or contributes to the comatose state.

Given the pharmacological profile of KYNA, the results suggest that glutamatergic and cholinergic neurotransmission are involved in the pathophysiology of CM. Persistent cognitive impairment is increasingly recognized as important sequelae of CM [3, 20]. NMDAR, as well as α7nAChR have a widely accepted role in cognitive function [12] and increased levels of brain KYNA have recently been shown to impair spatial working memory [41] and cognitive flexibility [11] in rats. Similarly, a reduction in endogenous KYNA enhances cognitive behaviour [42, 43]. The present study demonstrates for the first time that CSF kynurenine and KYNA are associated with impaired cognitive functioning in human CM. The association was only seen in children ≥5 years of age, even though children <5 years had higher levels of kynurenine and KYNA. The reason for the discrepancy is unclear, but could relate to differences in the tests used in the two age groups (i.e., the tests used in children ≥5 years of age may better assess functions affected by kynurenine or KYNA) or possibly a more powerful effect of kynurenine and KYNA in the more formed brain of the older child, who may have less neural plasticity to counteract the effects of KYNA, even at lower levels. CSF KYNA concentrations in Herpes Simplex encephalitis [15] and Tick-borne encephalitis (unpublished observation) can remain elevated for 6 months to a year after the episode of disease. If similar persistent elevation occurs in CM, KYNA could causally contribute to long-term neurocognitive impairment. In this regard, the kynurenine pathway may be a potential target for therapeutic interventions.

However, the pathophysiological significance of an activation of the kynurenine pathway as a result of an immune activation in encephalitis is unclear. It has been speculated that KYNA may suppress infection and prevent acute infection-related neurotoxicity [15], and KYNA has been shown to display a neuroprotective effect acutely, both experimentally [44] and clinically [15]. KYNA-related neuroprotection may be due to a counterbalance of the neurotoxic action of quinolinic acid, an NMDAR agonist, also forming part of the kynurenine pathway [10]. Thus, neurodegeneration by the excitotoxic kynurenine metabolite quinolinic acid in CM [19] may acutely be prevented by the simultaneous presence of the high levels of brain KYNA. However, persistent presence of KYNA could potentially induce long-lasting cognitive deficits by lowering neurotransmission in glutamatergic and cholinergic synapses. KYNA may also affect NMDAR mediated neuronal plasticity by preventing the formation of synaptic structure and function [45–47], and affect long-term cognition in this way. The dual actions of KYNA could complicate efforts for drug therapy targeting the kynurenine pathway, as a reduction in brain KYNA might be valuable for prevention of long-term neurocognitive impairment, but may, simultaneously, increase the risk of acute gross neurologic deficits.

## Conclusions

CSF KYNA, as well as its immediate precursor kynurenine, are elevated in children with CM and correlates to longer coma duration and long-term impairment in attention. Given the pharmacological profile of KYNA, i.e. an antagonist at the glycine site of the NMDAR as well as on the α7nAChR, elevation of brain KYNA is associated with a considerable impairment in glutamatergic and cholinergic signaling. This impairment may be causally related the development of coma that defines the acute phase of the disease as well the long-term neurocognitive impairment seen after recovery.

## Abbreviations

CNS: central nervous system
CM: cerebral malaria
CSF: cerebrospinal fluid
KYNA: kynurenic acid
NMDAR: *N*-methyl-d-aspartate-receptor
α7nAChR: cholinergic α7 nicotinic receptor
HIV: human immunodeficiency virus
HPLC: high pressure liquid chromatography
KABC: Kaufman assessment battery for children
CI: confidence interval
IFN: interferon
TNF: tumour necrosis factor
TDO: tryptophan 2,3-dioxygenase
IDO: indoleamine 2,3-dioxygenase
